# Endoscopic gallbladder-preserving cholecystolithotomy via a combined percutaneous and endoscopic ultrasound-guided approach

**DOI:** 10.1055/a-2808-7557

**Published:** 2026-03-05

**Authors:** Hui Zhang, Tao Wang, Zhiwei Jiang, Xiao Ma, Yi Wen

**Affiliations:** 1 26489 Department of General Surgery, The General Hospital of Western Theater Command, Chengdu, China

Endoscopic gallbladder-preserving cholecystolithotomy via endoscopic retrograde cholangiopancreatography often risks injury to the sphincter of Oddi and cystic duct spiral valves 1 2 3 . We report an elderly patient successfully managed with a “scarless” stone removal approach combining percutaneous transhepatic gallbladder drainage (PTGD) with an endoscopic ultrasound (EUS)-guided lumen-apposing metal stent (LAMS) placement, preserving these important structures.

**Fig. 1:**
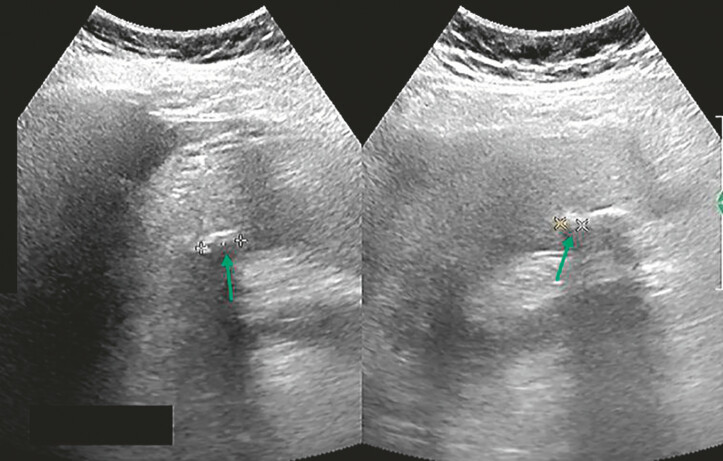
Abdominal ultrasound reveals multiple gallstones with cholecystitis in an 84-year-old female patient, measuring 0.8–1.2 cm in diameter. Arrows indicate the location of the stones.

**Fig. 2:**
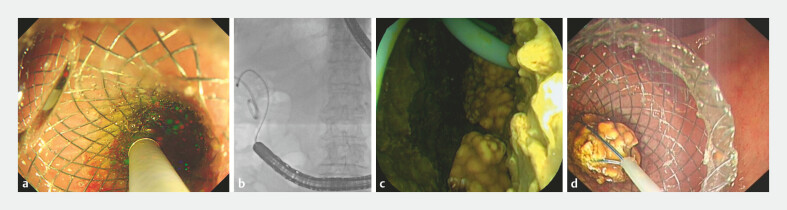
A peroral cholangioscope entering the gallbladder cavity for stone extraction. **a** Direct visualization during the insertion of the peroral cholangioscope. **b** A fluoroscopic view confirming the cholangioscope within the gallbladder cavity. **c** A direct endoscopic view showing the PTGD drainage catheter and several yellow gallstones inside the gallbladder. **d** Stone retrieval performed through the peroral cholangioscope. PTGD, percutaneous transhepatic gallbladder drainage.

**Fig. 3:**
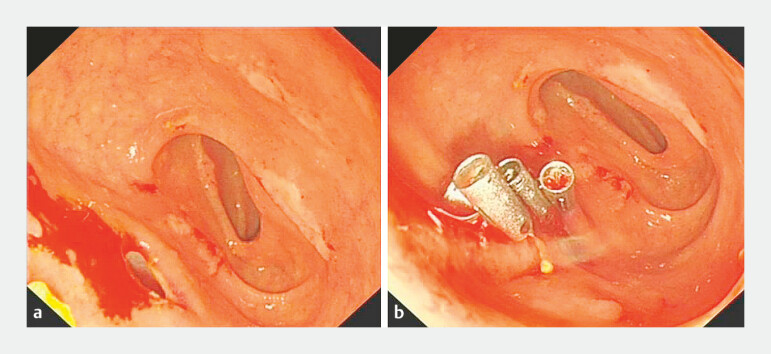
The endoscopic retrieval of the LAMS and closure of the duodenal bulb fistula. **a** Fistula opening at the duodenal bulb observed after the snare retrieval of the LAMS. **b** Closure of the fistula using four metallic clips. LAMS, lumen-apposing metal stent.

The patient was admitted due to cholecystolithiasis with cholecystitis ( Fig. 1 ). Owing to poor cardiopulmonary function which precluded laparoscopic cholecystectomy, PTGD was first performed, with the placement of a 7-French pigtail drainage catheter. During the subsequent endoscopic phase, an ultrasonic endoscope was used to visualize the gallbladder from the duodenal bulb while saline was infused via the PTGD catheter for optimal distention. Under Doppler monitoring, the gallbladder was punctured and a LAMS stent (15 × 10 mm) was deployed ( Video 1 ). After switching to a enteroscope, the LAMS was dilated using a balloon catheter (30 × 10 mm). A peroral cholangioscope was then introduced into the gallbladder lumen to remove the stones ( Fig. 2 ). After complete stone clearance, the gallbladder was irrigated via the PTGD catheter to flush residual stone fragments into the intestinal lumen, and irrigation was continued until no further stone fragments were observed to pass. Finally, the LAMS was retrieved using a snare, and the mucosal defect on the duodenal bulb was closed with four metallic clips ( Fig. 3 ).

PTGD with EUS-LAMS for gallbladder-preserving cholecystolithotomy. EUS-LAMS, endoscopic ultrasound-lumen-apposing metal stent PTGD, percutaneous transhepatic gallbladder drainage.Video 1

**Fig. 4:**
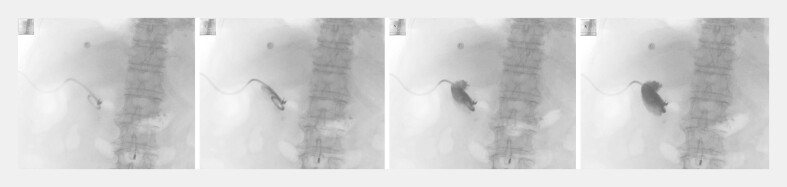
Cholecystography on postoperative day 7 demonstrating an intact gallbladder wall without contrast extravasation.

**Fig. 5:**
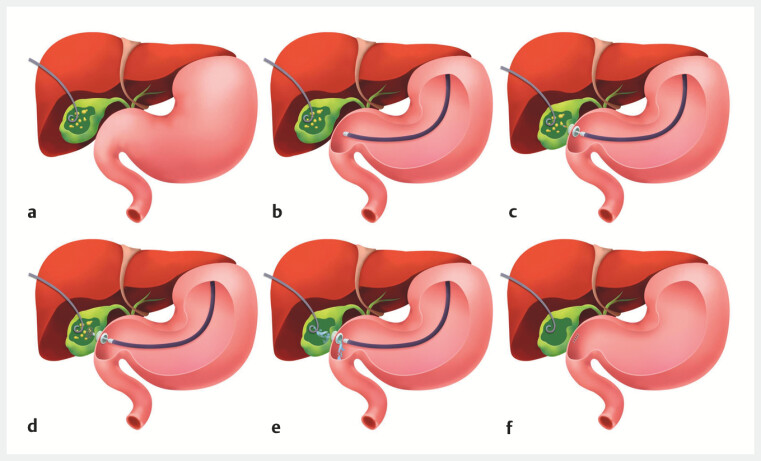
Schematic illustration of the procedural steps. **a** Percutaneous transhepatic gallbladder drainage (PTGD) with the placement of a pigtail catheter. **b** Endoscopic ultrasound (EUS) examination of the gallbladder from the duodenal bulb. **c** The EUS-guided placement of a LAMS. **d** Gallstone extraction through the LAMS. **e** Irrigation of the gallbladder via the PTGD catheter. **f** The retrieval of the LAMS right after stone extraction, followed by closure of the duodenal bulb fistula. The drainage catheter can be removed 1–2 weeks postoperatively after cholecystography confirms healing of the gallbladder wall defect. LAMS, lumen-apposing metal stent.

On postoperative day 7, cholecystography was performed via the PTGD catheter. The contrast study revealed an intact gallbladder wall with no evidence of contrast extravasation ( Fig. 4 ). Subsequently, the PTGD drainage catheter was removed. The combined approach can achieve early closure of the duodenal bulb defect and natural serosal healing of the gallbladder wall puncture site, thereby avoiding injury to the sphincter of Oddi and the cystic duct spiral valve ( Fig. 5 ). This technique is particularly suitable for elderly patients with higher surgical risks.

Endoscopy_UCTN_Code_TTT_1AS_2AK
